# Consistency as the Currency in Psychological Measures: A Reliability Generalization Meta-Analysis of Kessler Psychological Distress Scale (K-10 and K-6)

**DOI:** 10.1155/2024/3801950

**Published:** 2024-10-16

**Authors:** Ajele Kenni Wojujutari, Erhabor Sunday Idemudia

**Affiliations:** Faculty of Humanities, North-West University, Mafikeng, South Africa

**Keywords:** cultural adaptation, Kessler Psychological Distress Scale, meta-analysis, psychological distress, psychometrics, reliability

## Abstract

**Background:** Psychological distress is a critical concern in mental health, significantly impacting the quality of life across lifespan. Reliable and culturally adaptable assessment tools are essential for effective diagnosis and intervention. The Kessler Psychological Distress Scales (K-10 and K-6) are widely used for their efficiency and psychometric strength, but the reliability of K-10 and K-6 across different populations and settings remains to be determined.

**Objective:** This study aims to evaluate the reliability generalization (RG) of the K-10 and K-6 scales across diverse demographic and cultural contexts, providing a comprehensive meta-analysis of their performance.

**Method:** A RG meta-analysis was conducted using data from peer-reviewed articles published between 2002 and 2024, sourced from databases such as Web of Science, Scopus, and ScienceDirect. The analysis included 48 studies that reported reliability measures like Cronbach's *α*, focusing on the psychometric properties of the scales across various populations and settings.

**Results:** The meta-analysis revealed high internal consistency for both the K-10 (mean *α* = 0.90, 95% confidence interval (CI) [0.88, 0.91]) and K-6 (mean *α* = 0.84, 95% CI [0.80, 0.88]) scales. Reliability varied across different populations and languages. For the K-10, the highest reliability was found among adolescents (*α* = 0.93) and carers (*α* = 0.91). The K-10 demonstrated exceptional reliability in settings such as Australia (*α* = 0.97) and significant variability in Tanzania (*α* = 0.78). The K-6 scale showed high reliability among outpatients (*α* = 0.89) and the general population (*α* = 0.87). The scales were adapted into multiple languages, including English, Chinese, Swahili, Farsi, Indonesian, Japanese, Hindi, and Portuguese, reflecting their global applicability and adaptability.

**Conclusion:** The Kessler Psychological Distress Scales (K-10 and K-6) are reliable tools for measuring psychological distress in general and clinical populations. Their high reliability and adaptability across diverse settings highlight their value in clinical practice and research. These findings support the continued use and adaptation of these scales in global mental health assessments, emphasizing the importance of cultural and linguistic considerations.

## 1. Introduction

In the dynamic field of mental health, accurately measuring psychological distress stands as a cornerstone for both clinical diagnosis and epidemiological research. The high prevalence of anxiety and depression has raised psychological distress to a significant global public health issue, affecting diverse demographic and cultural groups [1–3]. In response to this ubiquitous challenge, assessment tools that transcend geographical and sociocultural barriers are indispensable for identifying, measuring, and subsequently facilitating the early intervention and treatment of psychological distress. The Kessler Psychological Distress Scale (K-10) and its abbreviated form (K-6) stand out for their efficiency, accessibility, and psychometric robustness, leading to their widespread use in diverse populations for assessing mental health [4].

Since their inception, the Kessler Psychological Distress Scales (K-10 and K-6) have been integral to global efforts in understanding and mitigating psychological distress, underpinning numerous studies and interventions [5]. The effectiveness of these measures depends on their reliability—consistency across time, observers, and contexts. This attribute is not merely a statistical convenience but the linchpin of an instrument's capacity to accurately capture the phenomena it aims to measure, thereby validating the conclusions drawn from its data [6, 7].

Studies from countries such as Japan, the United States, South Africa, Australia, and German-speaking countries have validated the Kessler Psychological Distress Scales (K-10 and K-6) as reliable tools across different cultural and linguistic settings, supporting their cross-cultural applicability in clinical and research contexts [8–13].

These findings collectively support the notion that the Kessler Psychological Distress Scale, both the K-10 and K-6 versions, exhibit high internal consistency across various populations, emphasizing its reliability for assessing psychological distress. Recent studies underscore the necessity of considering linguistic and cultural factors when using the Kessler Psychological Distress Scales, highlighting their adaptability and reliability [14–16].

Despite their extensive application in research and practice, a comprehensive synthesis of the K-10 and K-6 scales' reliability, examining their performance across different demographic and clinical populations, geographical locales, and administration conditions, remains conspicuously lacking. This reliability generalization (RG) meta-analysis aims to assess the consistency of the K-10 and K-6 scales across various studies. It provides comprehensive insights into their reliability and offers directions for future research and clinical applications. Through this comprehensive analysis, we aim to emphasize the crucial significance of reliability in assessing psychological distress, thereby enhancing the accuracy and importance of these measures in both mental health research and clinical settings.

## 2. Methods

### 2.1. Design

The methods utilized in our study included a RG meta-analysis aimed at a comprehensive evaluation of the psychometric properties of the Kessler Psychological Distress Scales (K-10 and K-6). To ensure maximum transparency and credibility, we preregistered the study protocol on Prospero with the registration number CRD42024515783. By implementing preregistration, we aimed to promote transparency, mitigate potential biases, and counteract biases in the research design and the analysis.

### 2.2. Search Strategy

The meta-analysis on the RG of the Kessler Psychological Distress Scales (K-10 and K-6) utilized a comprehensive search approach covering well-known databases such as Web of Science, Scopus, ScienceDirect, Google Scholar, and EBSCOhost. Searches were conducted between 2002 and 2024, focusing on peer-reviewed journals in English.

To pinpoint the most relevant studies, a combination of keywords such as “Kessler Psychological Distress Scale K-10,” “K-10,” and Kessler “Psychological Distress Scale K-6,” “K-6,” “Psychological Distress Scale,” “reliability,” “psychometrics properties,” “validity,” “internal consistency,” and “test-retest reliability” was employed. Moreover, the amalgamation of keywords with Boolean operators in the search strategy, such as (“Kessler Psychological Distress Scale K-10,” OR “Kessler Psychological Distress Scale” OR “K-10” OR “Kessler Psychological Distress Scale K-6,” OR “K-6”) AND (“reliability” OR “internal consistency” OR “test-retest reliability” OR “psychometrics properties”) was utilized.

We used this methodology to ensure a thorough analysis of the literature concerning the reliability of the Kessler Psychological Distress Scales (K-10 and K-6) across different demographics, environments, and research methodologies, including both published and gray literature. Manual perusal of bibliographies and direct contact with authors supplemented database queries to uncover further pertinent studies.

### 2.3. Criteria for Inclusion and Exclusion

The present systematic review evaluates the reliability of the Kessler Psychological Distress Scales (K-10 and K-6) in measuring psychological distress throughout different life phases, including adolescence, adulthood, the general population, and the clinical population. This review selected articles based on strict criteria to assess the reliability of the Kessler Psychological Distress Scales (K-10 and K-6). Specifically, peer-reviewed articles presented reliability measures such as Cronbach's *α* and test–retest reliability, focusing on the psychometric properties of the K-10 and K-6 scales across various populations and versions. This selection process focused on gaining insights into the scale's reliability across diverse contexts, specifically emphasizing English-language articles for consistency. Exclusion criteria were employed to eliminate nonresearch articles, gray literature, non-peer-reviewed sources, and publications in languages other than English to enhance clarity and uphold the review's integrity. This systematic approach facilitated the inclusion of studies that offer valuable insights into the reliability of the K-10 and K-6 scales, thereby strengthening evidence-based clinical practice for psychological-related distress.

### 2.4. Data Extraction Process

We meticulously extracted the data to confidently and robustly perform the meta-analysis. We assessed the reliability of the Kessler Psychological Distress Scales (K-10 and K-6) in each study and deliberated discrepancies to establish a solid analytical base. Essential attributes of the research, such as authorship, publication date, study methodology, version and language of K-10 and K-6 scales, Cronbach's *α*, item quantity, population demographics, country, continent, and sample size, were meticulously documented. These details played a crucial role in evaluating study quality and context. Reliability measures, including Cronbach's *α*, were extracted to evaluate the consistency and stability of the K-10 and K-6 scales. We also considered participant demographics and clinical profiles to assess the relevance of the reliability findings. Recording methodological details, such as techniques for assessing reliability, data collection procedures, and statistical analysis methods, contributed to assessing study rigor and validating reliability evaluations. This systematic approach ensured a credible synthesis of evidence concerning the reliability of the K-10 and K-6 scales in our meta-analysis.

### 2.5. Evaluation of Publication Bias and Quality

We dedicated meticulous attention to addressing publication bias and quality assessment to uphold the credibility and validity of our results. The team conducted a thorough investigation across numerous databases, identifying publication bias using funnel plot analysis and applying necessary modifications with the trim and fill method. We used the COSMIN Checklist and QUADAS-2 to evaluate the methodological quality, following the guidelines [17, 18]. This methodology scrutinized study attributes and statistical approaches to ensure a consistent and comprehensive utilization of the Kessler Psychological Distress Scales (K-10 and K-6). Our objective was to present a thorough and dependable synthesis of evidence regarding the reliability of the Kessler Psychological Distress Scales (K-10 and K-6), which would contribute to enhancing research and clinical practices in the management of psychological-related distress.

### 2.6. Data Analysis

An extensive examination of data was conducted on the reliability of the Kessler Psychological Distress Scales (K-10 and K-6) through meta-analysis using R-Studio software, employing a systematic approach to amalgamate research findings. We used customized R scripts to extract key reliability metrics, such as Cronbach's *α*, from various studies to ensure consistent data gathering. The meta-analysis utilized statistical packages like “meta” and “metaphor,” implementing random-effect models to aggregate reliability measures while accounting for variations in study characteristics.

To offer a detailed perspective on the reliability of the Kessler Psychological Distress Scales (K-10 and K-6), summary statistics, including combined reliability estimates and confidence intervals (CIs) at a 95% level, were computed. We evaluated heterogeneity using metrics like the *Q* and *I*^2^ statistics, then conducted subgroup and sensitivity analyses to identify sources of diversity among the studies and validate the robustness of our results.

Furthermore, R-Studio software facilitated the assessment of publication bias via funnel plot analysis and conducted sensitivity analyses to evaluate the potential influence of biases. We performed subgroup analyses to investigate variations in reliability estimates across different populations, versions of the Kessler Psychological Distress Scales (K-10 and K-6), and languages. The utilization of visualization tools in R aided in interpreting these analyses. Overall, the extensive functionalities of R ensured a thorough and credible meta-analysis, providing valuable insights into the reliability of the Kessler Psychological Distress Scales (K-10 and K-6) in the context of clinical practice and research.

## 3. Results

### 3.1. Reliability Report Inducement of the Included Studies

**Figure 1:**
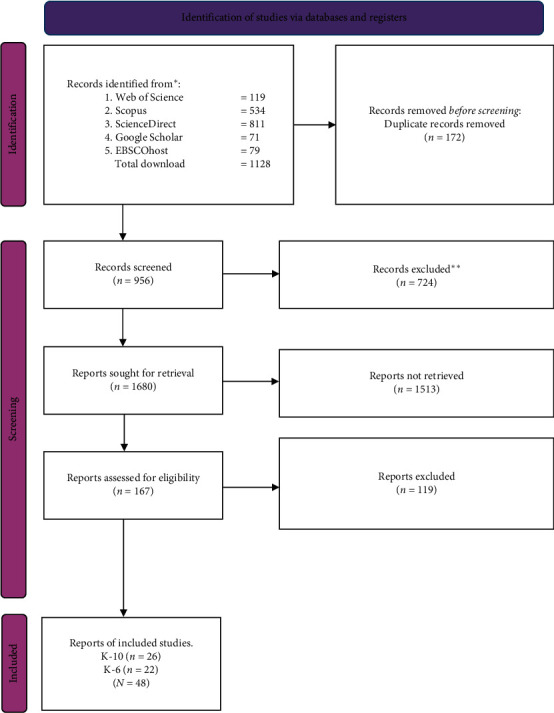
PRISMA 2020 flow diagram of the studies selection process. The symbol “*⁣*^*∗*^” indicates that the studies were identified from multiple databases, as listed in the figure (e.g., Web of Science, Scopus, ScienceDirect, etc.); “*⁣*^*∗∗*^” signifies records that were excluded after the initial screening process, which may be due to reasons, such as irrelevance to the research question, lack of access to full texts, or other criteria for exclusion, as described in the methodology; and “*⁣*^*∗∗∗*^” is used to mark reports excluded after the final eligibility assessment, based on predefined exclusion criteria, such as not meeting the required quality standards or relevance to the research topic.

The meticulous selection process, as depicted in the preferred reporting items for systematic reviews and meta-analyses (PRISMA) 2020 flowchart, commenced with a search across multiple databases, including Web of Science, Scopus, ScienceDirect, Google Scholar, and EBSCOhost, yielding 1128 records (Figure 1). We removed 172 duplicates and screened 956 records, ultimately selecting 145 reports for retrieval. Challenges in retrieval reduced this number to 167 reports assessed for eligibility, with 48 studies meeting the rigorous inclusion criteria of 236,259 participants. This meticulous process underscores the diligence in ensuring the reliability and validity of the Kessler Psychological Distress Scales (K-10 and K-6) across different settings.

Data regarding authors, publication year, sample size, scale items, Cronbach's *α*, population characteristics, country, language adaptation, and continent were extracted from these studies, spanning from 2002 to 2024. The studies showcased a broad range of sample sizes, from small groups to large cohorts, illustrating the scales' versatility. Cronbach's *α* values across the studies spanned from 0.76 to 0.97, demonstrating high internal consistency and reliability across different adaptations of the scales. The K-10 scale exhibited exceptional reliability among Australian adolescents (*α* = 0.97), marking significant efficacy in youth mental health evaluations. In contrast, the lowest reliability reported was with the K-6 scale in a Tanzanian healthcare setting (*α* = 0.78), highlighting potential cultural or linguistic adaptation challenges.

The scales have been adapted into several languages, including English, Chinese, Swahili, Farsi, Indonesian, Japanese, Hindi, and Portuguese, reflecting their global applicability and adaptability to diverse cultural contexts. The geographical distribution of the included studies spanned continents such as Asia, Africa, North America, Australia, and South America, illustrating the scales' utility across different healthcare settings, from general population surveys to specific demographic groups like immigrants, outpatients, and adolescents. This wide-ranging application underscores the necessity of careful adaptation and validation processes to maintain reliability across diverse linguistic and cultural settings.

This synthesis underscores the Kessler Psychological Distress Scales' consistent reliability and adaptability across various settings, highlighting the importance of cultural and linguistic adaptation in mental health assessment tools. The demonstrated utility of these scales in assessing psychological distress across varied populations affirms their value in international mental health research and clinical practice. Future research should aim to elucidate factors contributing to variability in scale reliability to enhance global applicability, fostering the development of cultural and linguistic modification guidelines.

The systematic review confirms that the Kessler Psychological Distress Scales are reliable global mental health assessment tools adaptable across a broad spectrum of linguistic and cultural settings. The findings from this synthesis provide an important contribution to the field, suggesting directions for future research to enhance the scales' utility worldwide.

### 3.2. K-10 Meta-Analysis of RG

**Figure 2:**
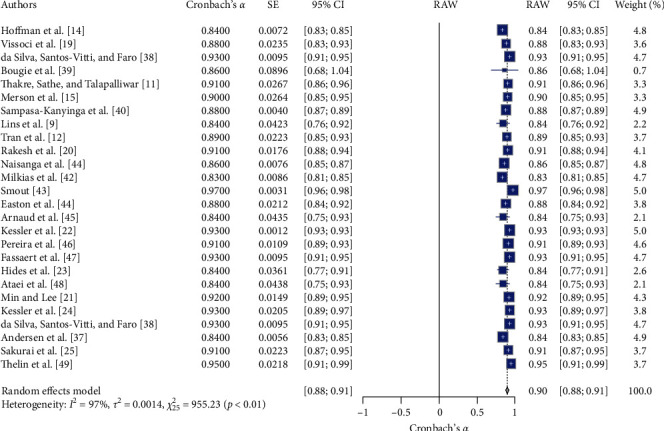
The forest plot for K-10 reliability estimate across included studies. *Note:* This figure presents the forest plot for K-10 reliability estimates across the included studies. The figure provides meta-analysis results, including the estimated reliability (Cronbach's *α*), standard error (SE), 95% confidence intervals (95% CI), the weight of each study in the random effects model, and the overall mean reliability (RAW). The random effects model indicates significant reliability estimates with substantial heterogeneity. Square symbols represent the Cronbach's *α* reliability estimate for each individual study included in the meta-analysis. Horizontal lines indicate the 95% confidence intervals (CIs) for each study's reliability estimate. Diamond symbol at the bottom represents the overall pooled Cronbach's *α* reliability estimate across all studies in the random effects model, with its width corresponding to the 95% confidence interval. Size of squares reflects the weight of each study in the random effects model, with larger squares indicating studies that contributed more to the pooled estimate.

**Figure 3:**
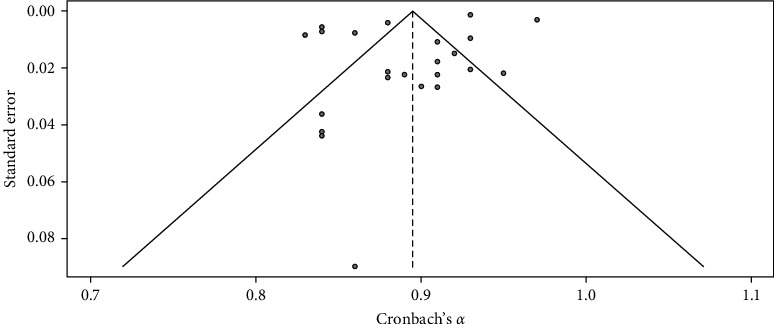
Funnel plot for K-10 reliability estimate across included studies. *Note:* This figure presents the funnel plot for K-10 reliability estimates across the included studies. The funnel plot is used to assess the presence of publication bias in the meta-analysis. The distribution of the studies around the overall mean reliability suggests minimal publication bias.

Table 1 and Figures 2 and 3 show the meta-analysis results, which aimed to evaluate the RG of the K-10 scale, a measure used to assess psychological distress levels. The meta-analysis of the K-10 scale's RG involved 26 studies. The random effects model indicated high overall reliability for the K-10 scale, with a mean Cronbach's *α* of 0.90, 95% CI [0.88, 0.91], *z* = 106.55, *p*  < 0.01. The heterogeneity was substantial (*I*^2^ = 97.4%, *Q* (25) = 955.23, *p*  < 0.05), suggesting significant variability in the reliability estimates across studies. The funnel plot (see Figure 1) did not show significant asymmetry, indicating minimal publication bias. The RG plot (see Figure 2) illustrates Cronbach's *α* value distribution across the studies. The *H* statistic further quantifies this heterogeneity (*H* = 7.6.18, 95% CI [5.61, 6.81]), highlighting the variability in reliability estimates across the included studies.

### 3.3. Meta-Analysis of Population Characteristics and Reliability of K-10

**Figure 4:**
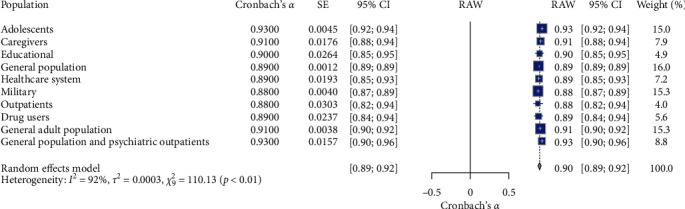
Forest plot for K-10 reliability estimate on population characteristics. *Note:* This figure presents the forest plot for K-10 reliability estimates across different population characteristics. The figure provides meta-analysis results, including the estimated reliability (Cronbach's *α*), standard error (SE), 95% confidence intervals (95% CI), the weight of each population subgroup in the random effects model, and the overall mean reliability (RAW). The random effects model indicates significant reliability estimates with substantial heterogeneity. Square symbols represent the Cronbach's *α* reliability estimate for each individual study included in the meta-analysis. Horizontal lines indicate the 95% confidence intervals (CIs) for each study's reliability estimate. Diamond symbol at the bottom represents the overall pooled Cronbach's *α* reliability estimate across all studies in the random effects model, with its width corresponding to the 95% confidence interval. Size of squares reflects the weight of each study in the random effects model, with larger squares indicating studies that contributed more to the pooled estimate.

Table 1 and Figure 4 present the meta-analysis of the K-10 scale's reliability across different population subgroups involving 10 studies (*N* = 10). The random effects model indicated high overall reliability for the K-10 scale, with a mean Cronbach's *α* of 0.90, 95% CI [0.89, 0.92], *z* = 129.48, *p*  < 0.01. Adolescents exhibited the highest reliability coefficient (*α* = 0.93, 95% CI [0.92, 0.94]), indicating very high consistency. Caregivers showed a reliability coefficient of 0.91 (95% CI [0.88, 0.94]), suggesting high reliability. The educational population had a mean reliability coefficient of 0.90 (95% CI [0.85, 0.95]), reflecting good reliability. The general population presented a mean reliability coefficient of 0.89 (95% CI [0.89, 0.89]), demonstrating consistency. The healthcare system reported a reliability coefficient of 0.89 (95% CI [0.85, 0.93]). The military subgroup showed a reliability coefficient of 0.88 (95% CI [0.87, 0.89]), with the largest weight in the common effect model (15.3%), suggesting high consistency. Outpatients exhibited a reliability coefficient of 0.88 (95% CI [0.82, 0.94]), with more significant variability. Drug users had a mean reliability coefficient of 0.89 (95% CI [0.84, 0.94]). The general adult population showed a reliability coefficient of 0.91 (95% CI [0.90, 0.92]). The general population and psychiatric outpatient subgroup reported a high-reliability coefficient of 0.93 (95% CI [0.90, 0.96]). Significant heterogeneity was observed (*I*^2^ = 91.8%, *τ*^2^ = 0.0003), as indicated by the *Q* test (*Q* = 110.13, df = 9, *p*  < 0.05). The RG plot (see Figure 4) illustrates the distribution of Cronbach's *α* values across the subgroups.

### 3.4. Meta-Analysis K-10 of Language and Adaptation Reliability

**Figure 5:**
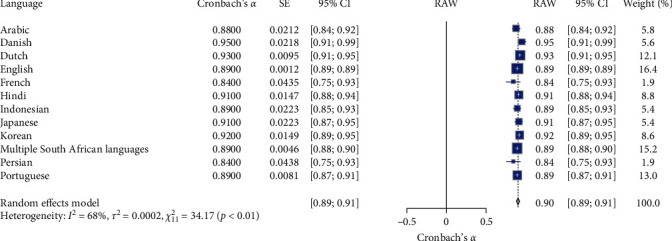
Forest for K-10 reliability estimate on language and adaptation. *Note:* This figure presents the forest plot for K-10 reliability estimates across different language and adaptation subgroups. The figure provides meta-analysis results, including the estimated reliability (Cronbach's *α*), standard error (SE), 95% confidence intervals (95% CI), the weight of each language subgroup in the random effects model, and the overall mean reliability (RAW). The random effects model indicates significant reliability estimates with substantial heterogeneity. Square symbols represent the Cronbach's *α* reliability estimate for each individual study included in the meta-analysis. Horizontal lines indicate the 95% confidence intervals (CIs) for each study's reliability estimate. Diamond symbol at the bottom represents the overall pooled Cronbach's *α* reliability estimate across all studies in the random effects model, with its width corresponding to the 95% confidence interval. Size of squares reflects the weight of each study in the random effects model, with larger squares indicating studies that contributed more to the pooled estimate.

Table 1 and Figure 5 present the meta-analysis results of the Kessler Psychological Distress Scale (K-10) reliability across different languages and cultural adaptations. The meta-analysis of the K-10 scale's reliability involved 12 language and cultural adaptation subgroups (*N* = 12). The random effects model indicated a high overall reliability for the K-10 scale, with a mean Cronbach's *α* of 0.90, 95% CI [0.89, 0.91], *z* = 141.90, *p*  < 0.001. Significant heterogeneity was observed (*I*^2^ = 67.8%, *Q* (11) = 34.17, *p*  < 0.01), suggesting substantial variability in the reliability estimates across different languages and cultural adaptations. Specifically, the reliability coefficients varied across different languages: Arabic had a reliability coefficient of 0.88 (95% CI [0.84, 0.92]); Danish had the highest reliability coefficient of 0.95 (95% CI [0.91, 0.99]); Dutch had a reliability coefficient of 0.93 (95% CI [0.91, 0.95]); English had a reliability coefficient of 0.89 (95% CI [0.89, 0.89]); French had the lowest reliability coefficient of 0.84 (95% CI [0.75, 0.93]); Hindi had a reliability coefficient of 0.91 (95% CI [0.88, 0.94]); Indonesian had a reliability coefficient of 0.89 (95% CI [0.85, 0.93]); Japanese had a reliability coefficient of 0.91 (95% CI [0.87, 0.95]); Korean had a reliability coefficient of 0.92 (95% CI [0.89, 0.95]); Multiple South African Languages had a reliability coefficient of 0.89 (95% CI [0.88, 0.90]); Persian had a reliability coefficient of 0.84 (95% CI [0.75, 0.93]); and Portuguese had a reliability coefficient of 0.89 (95% CI [0.87, 0.91]). These results suggest that the K-10 scale demonstrates high reliability across various languages and cultural adaptations, with Danish showing the highest reliability and French the lowest. Significant heterogeneity indicates variability in reliability estimates across different adaptations. The RG plot (see Figure 5) illustrates Cronbach's *α* values distribution across the subgroups.

### 3.5. Meta-Analysis K-10 Continent Subgroups Reliability

**Figure 6:**
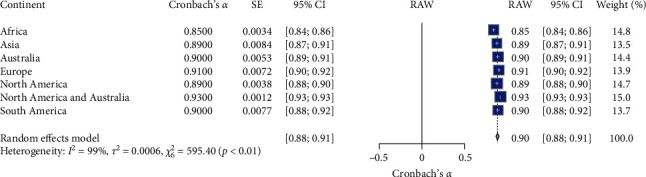
Forest for K-10 reliability estimate on continents. *Note:* This figure presents the forest plot for K-10 reliability estimates across different continents. The figure provides meta-analysis results, including the estimated reliability (Cronbach's *α*), standard error (SE), 95% confidence intervals (95% CI), the weight of each continent subgroup in the random effects model, and the overall mean reliability (RAW). The random effects model indicates significant reliability estimates with substantial heterogeneity. Square symbols represent the Cronbach's *α* reliability estimate for each individual study included in the meta-analysis. Horizontal lines indicate the 95% confidence intervals (CIs) for each study's reliability estimate. Diamond symbol at the bottom represents the overall pooled Cronbach's *α* reliability estimate across all studies in the random effects model, with its width corresponding to the 95% confidence interval. Size of squares reflects the weight of each study in the random effects model, with larger squares indicating studies that contributed more to the pooled estimate.

Table 1 and Figure 6 present the meta-analysis results of the Kessler Psychological Distress Scale (K-10) reliability across different continents. The meta-analysis of the K-10 scale's reliability across different continent subgroups (*N* = 7). The random effects model indicated high overall reliability for the K-10 scale, with a mean Cronbach's *α* of 0.90, 95% CI [0.88, 0.91], *z* = 93.78, *p*  < 0.05. Significant heterogeneity was observed (*I*^2^ = 99.0%, *Q* (6) = 595.40, *p*  < 0.05), suggesting substantial variability in the reliability estimates across different continents. Specifically, the reliability coefficients varied across different continents: Africa had a reliability coefficient of 0.85 (95% CI [0.84, 0.86]); Asia had a reliability coefficient of 0.89 (95% CI [0.87, 0.91]); Australia had a reliability coefficient of 0.90 (95% CI [0.89, 0.91]); Europe had a reliability coefficient of 0.91 (95% CI [0.90, 0.92]); North America had a reliability coefficient of 0.89 (95% CI [0.88, 0.90]); North America and Australia combined had the highest reliability coefficient of 0.93 (95% CI [0.93, 0.93]); and South America had a reliability coefficient of 0.90 (95% CI [0.88, 0.92]). These results suggest that the K-10 scale demonstrates high reliability across various continents, with North America and Australia combined showing the highest reliability. Significant heterogeneity indicates variability in reliability estimates across different continents. The RG plot (see Figure 6) illustrates Cronbach's *α* value distribution across the subgroups.

### 3.6. K-6 Meta-Analysis of RG

**Figure 7:**
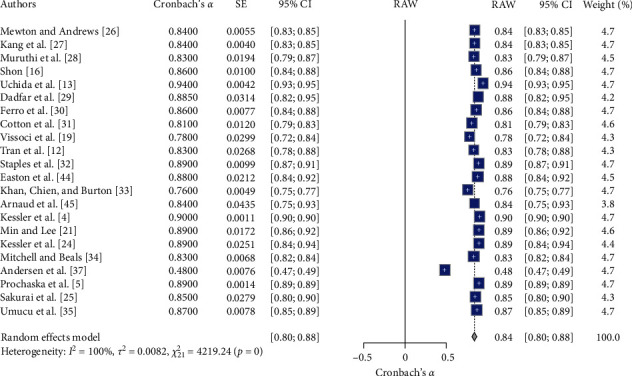
Forest plot for K-6 reliability estimate across included studies. *Note:* This figure presents the forest plot for K-6 reliability estimates across the included studies. The figure provides meta-analysis results, including the estimated reliability (Cronbach's *α*), standard error (SE), 95% confidence intervals (95% CI), the weight of each study in the random effects model, and the overall mean reliability (RAW). The random effects model indicates significant reliability estimates with substantial heterogeneity. Square symbols represent the Cronbach's *α* reliability estimate for each individual study included in the meta-analysis. Horizontal lines indicate the 95% confidence intervals (CIs) for each study's reliability estimate. Diamond symbol at the bottom represents the overall pooled Cronbach's *α* reliability estimate across all studies in the random effects model, with its width corresponding to the 95% confidence interval. Size of squares reflects the weight of each study in the random effects model, with larger squares indicating studies that contributed more to the pooled estimate.

**Figure 8:**
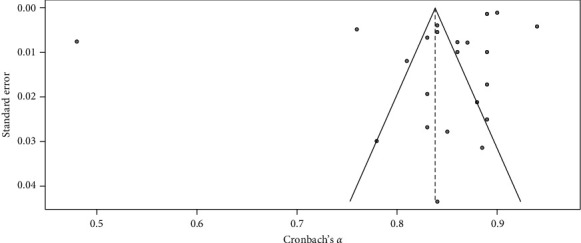
Funnel plot for K-6 reliability estimate across included studies. *Note:* This figure presents the funnel plot for K-6 reliability estimates across the included studies. The funnel plot is used to assess the presence of publication bias in the meta-analysis. The distribution of the studies around the overall mean reliability suggests minimal publication bias.

Table 2 and Figures 7 and 8 present the results of the comprehensive analysis of the RG of the Kessler Psychological Distress Scale (K-6). The meta-analysis of the K-6 scale's reliability involved 22 studies (*N* = 22). The random effects model indicated high overall reliability for the K-6 scale, with a mean Cronbach's *α* of 0.84, 95% CI [0.80, 0.88], *z* = 42.53, *p*  < 0.01. Significant heterogeneity was observed (*I*^2^ = 99.5%, *Q* (21) = 4219.24, *p*  < 0.05), suggesting substantial variability in the reliability estimates across different studies. These results suggest that the K-6 scale demonstrates high reliability across various studies, with significant heterogeneity indicating variability in reliability estimates. The funnel plot (Figure 8) did not show substantial asymmetry, indicating minimal publication bias. The forest plot (Figure 7) illustrates Cronbach's *α* value distribution across the included studies.

### 3.7. Meta-Analysis of Population Characteristics and Reliability of K-6

**Figure 9:**
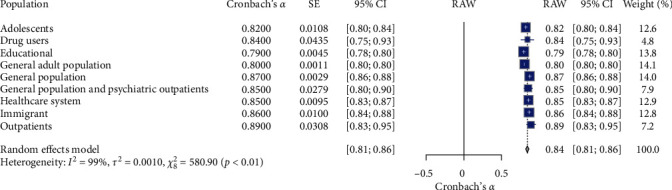
Forest plot for K-6 reliability estimate on population characteristics and reliability. *Note:* This figure presents the forest plot for K-6 reliability estimates across different population characteristics. The figure provides meta-analysis results, including the estimated reliability (Cronbach's *α*), standard error (SE), 95% confidence intervals (95% CI), the weight of each population subgroup in the random effects model, and the overall mean reliability (RAW). The random effects model indicates significant reliability estimates with substantial heterogeneity. Square symbols represent the Cronbach's *α* reliability estimate for each individual study included in the meta-analysis. Horizontal lines indicate the 95% confidence intervals (CIs) for each study's reliability estimate. Diamond symbol at the bottom represents the overall pooled Cronbach's *α* reliability estimate across all studies in the random effects model, with its width corresponding to the 95% confidence interval. Size of squares reflects the weight of each study in the random effects model, with larger squares indicating studies that contributed more to the pooled estimate.

Table 2 and Figure 9 present the meta-analysis results assessing the reliability of the Kessler Psychological Distress Scale (K-6) among diverse population subgroups. The meta-analysis of the K-6 scale's reliability across different population subgroups (*N* = 9). The random effects model indicated high overall reliability for the K-6 scale, with a mean Cronbach's *α* of 0.84, 95% CI [0.81, 0.86], *z* = 71.03, *p*  < 0.01. Significant heterogeneity was observed (*I*^2^ = 98.6%, *Q* (8) = 580.90, *p*  < 0.01), suggesting substantial variability in the reliability estimates across different subgroups. Specifically, the reliability coefficients varied across different population subgroups: Adolescents had a reliability coefficient of 0.82 (95% CI [0.80, 0.84]); drug users had a reliability coefficient of 0.84 (95% CI [0.75, 0.93]); educational population had a reliability coefficient of 0.79 (95% CI [0.78, 0.80]); general adult population had a reliability coefficient of 0.80 (95% CI [0.80, 0.80]); general population had a reliability coefficient of 0.87 (95% CI [0.86, 0.88]); general population and psychiatric outpatients had a reliability coefficient of 0.85 (95% CI [0.80, 0.90]); healthcare system had a reliability coefficient of 0.85 (95% CI [0.83, 0.87]); immigrant population had a reliability coefficient of 0.86 (95% CI [0.84, 0.88]); and outpatients had the highest reliability coefficient of 0.89 (95% CI [0.83, 0.95]). These results suggest that the K-6 scale demonstrates high reliability across various population subgroups, with significant heterogeneity indicating variability in reliability estimates. The forest plot (Figure 9) illustrates the Cronbach's *α* values distribution across the population subgroups.

### 3.8. Meta-Analysis of K-6 Language and Adaptation Reliability

**Figure 10:**
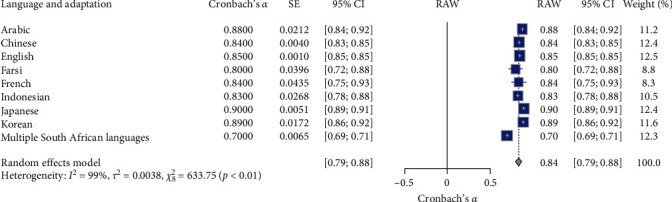
Forest plot for K-6 reliability estimate on language and adaptation. *Note:* This figure presents the forest plot for K-6 reliability estimates across different language and adaptation subgroups. The figure provides meta-analysis results, including the estimated reliability (Cronbach's *α*), standard error (SE), 95% confidence intervals (95% CI), the weight of each language subgroup in the random effects model, and the overall mean reliability (RAW). The random effects model indicates significant reliability estimates with substantial heterogeneity. Square symbols represent the Cronbach's *α* reliability estimate for each individual study included in the meta-analysis. Horizontal lines indicate the 95% confidence intervals (CIs) for each study's reliability estimate. Diamond symbol at the bottom represents the overall pooled Cronbach's *α* reliability estimate across all studies in the random effects model, with its width corresponding to the 95% confidence interval. Size of squares reflects the weight of each study in the random effects model, with larger squares indicating studies that contributed more to the pooled estimate.

Table 2 and Figure 10 present the meta-analysis results assessing the reliability of the Kessler Psychological Distress Scale (K-6) among diverse language and adaptation subgroups. The meta-analysis of the K-6 scale's reliability across different language and adaptation subgroups (*N* = 9). The random effects model indicated high overall reliability for the K-6 scale, with a mean Cronbach's *α* of 0.84, 95% CI [0.79, 0.88], *z* = 38.41, *p*  < 0.01. Significant heterogeneity was observed (*I*^2^ = 98.7%, *Q* (8) = 633.75, *p*  < 0.01), suggesting substantial variability in the reliability estimates across different language and adaptation subgroups. Specifically, the reliability coefficients varied across different languages and adaptations: Arabic had a reliability coefficient of 0.88 (95% CI [0.84, 0.92]); Chinese had a reliability coefficient of 0.84 (95% CI [0.83, 0.85]); English had a reliability coefficient of 0.85 (95% CI [0.85, 0.85]); Farsi had a reliability coefficient of 0.80 (95% CI [0.72, 0.88]); French had a reliability coefficient of 0.84 (95% CI [0.75, 0.93]); Indonesian had a reliability coefficient of 0.83 (95% CI [0.78, 0.88]); Japanese had the highest reliability coefficient of 0.90 (95% CI [0.89, 0.91]); Korean had a reliability coefficient of 0.89 (95% CI [0.86, 0.92]); and Multiple South African Languages had the lowest reliability coefficient of 0.70 (95% CI [0.69, 0.71]). These results suggest that the K-6 scale demonstrates high reliability across various languages and adaptations, with significant heterogeneity indicating variability in reliability estimates. The forest plot (Figure 10) illustrates Cronbach's *α* value distribution across the language and adaptation subgroups.

### 3.9. Meta-Analysis of K-6 Continent Reliability

**Figure 11:**
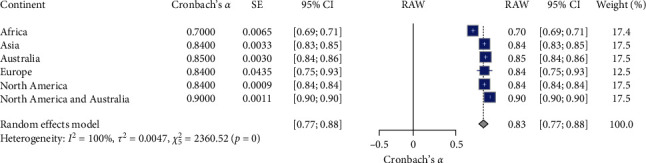
Forest plot for K-6 reliability estimate across different continents. *Note:* This figure presents the forest plot for K-10 reliability estimates across different continents. The figure provides meta-analysis results, including the estimated reliability (Cronbach's *α*), standard error (SE), 95% confidence intervals (95% CI), the weight of each continent subgroup in the random effects model, and the overall mean reliability (RAW). The random effects model indicates significant reliability estimates with substantial heterogeneity. Square symbols represent the Cronbach's *α* reliability estimate for each individual study included in the meta-analysis. Horizontal lines indicate the 95% confidence intervals (CIs) for each study's reliability estimate. Diamond symbol at the bottom represents the overall pooled Cronbach's *α* reliability estimate across all studies in the random effects model, with its width corresponding to the 95% confidence interval. Size of squares reflects the weight of each study in the random effects model, with larger squares indicating studies that contributed more to the pooled estimate.

Table 2 and Figure 11 present the meta-analysis results, revealing variations in the K-6 scale's reliability across different continents. The meta-analysis of the K-6 scale's reliability across different continent subgroups involved six studies (*N* = 6). The random effects model indicated a high overall reliability for the K-6 scale, with a mean Cronbach's *α* of 0.83, 95% CI [0.77, 0.88], *z* = 28.92, *p*  < 0.0001. Significant heterogeneity was observed (*I*^2^ = 99.8%, *Q* (5) = 2360.52, *p*  < 0.01), suggesting substantial variability in the reliability estimates across different continents. Specifically, the reliability coefficients varied across different continents: Africa had the lowest reliability coefficient of 0.70 (95% CI [0.69, 0.71]); Asia had a reliability coefficient of 0.84 (95% CI [0.83, 0.85]); Australia had a reliability coefficient of 0.85 (95% CI [0.84, 0.86]); Europe had a reliability coefficient of 0.84 (95% CI [0.75, 0.93]); North America had a reliability coefficient of 0.84 (95% CI [0.84, 0.84]); and North America and Australia combined had the highest reliability coefficient of 0.90 (95% CI [0.90, 0.90]). These results suggest that the K-6 scale demonstrates high reliability across various continents, with North America and Australia combined showing the highest reliability and Africa showing the lowest. Significant heterogeneity indicates variability in reliability estimates across different continents. The forest plot (Figure 11) illustrates Cronbach's *α* value distribution across the continent subgroups.

### 3.10. Comparative Meta-Analysis of RG for K-10 and K-6 Scales

Table 3 presents the results of the combined meta-analysis of the Kessler Psychological Distress Scales (K-10 and K-6). Descriptive statistics show that the mean Cronbach's *α* for K-10 is 0.89 (SD [standard deviation] = 0.04), and for K-6 is 0.84 (SD = 0.09). An independent sample *t*-test was conducted to compare the mean reliability coefficients (Cronbach's *α*) of the K-10 and K-6 scales. There was a significant difference in the mean reliability coefficients between K-10 (*M* = 0.89, SD = 0.04) and K-6 (*M* = 0.84, SD = 0.09); *t* (28.182) = 2.5036, *p*=0.01836, 95% CI [0.0095, 0.0945]. Cohen's *d* was calculated to measure the effect size of the difference in reliability coefficients between the K-10 and K-6 scales. The effect size was medium (*d* = 0.77, 95% CI [0.17, 1.35], indicating that the difference in reliability is practically significant).

## 4. Discussion

The RG meta-analysis of the Kessler Psychological Distress Scales (K-10 and K-6) presents compelling evidence of these tools' robustness and adaptability across diverse populations and contexts. The reported high-reliability coefficients underscore the scales' utility in global mental health assessments, highlighting their essential role in clinical practice and research [4, 7]. However, the analysis also uncovers significant heterogeneity among the included studies, highlighting critical nuances that warrant further discussion.

This heterogeneity suggests that while the scales exhibit high reliability, factors such as population characteristics, cultural context, and language adaptation significantly influence their performance. For instance, studies have shown that the reliability of the K-6 scale can vary when adapted to different languages, with some adaptations maintaining higher consistency than others [8, 36]. This variability underscores the need for rigorous local validation studies to ensure that adapted K-10 and K-6 scales accurately reflect the constructs they aim to measure in different cultural settings. Practical steps for local validation include collaboration with local researchers and incorporating culturally specific items into the scales. Andersen et al.'s [37] study reported a lower Cronbach's *α*, likely due to the small sample size and specific characteristics of the studied population. These factors emphasize the variability in K-10 reliability across different contexts and the importance of interpreting these metrics with caution.

Moreover, the comparative analysis between the K-10 and K-6 scales reveals a slightly higher reliability for the K-10, suggesting a potential trade-off between the brevity of the K-6 and the comprehensiveness and slightly superior reliability of the K-10. This finding aligns with previous research indicating that shorter scales, while beneficial for reducing respondent burden, may sometimes compromise the assessed construct's depth and granularity [6]. This implies that the K-10 may be preferable in settings where a more thorough assessment is required, while the K-6 could be used for quick screenings or in situations where time constraints are significant.

The observed differences in reliability across subgroups and languages also raise important considerations for future research. Studies included in the meta-analysis demonstrate variability in the scales' reliability among different demographic groups, such as adolescents versus older adults or clinical versus nonclinical populations [15, 16]. These findings suggest that demographic factors, including age and clinical status, may impact the scales' reliability. Future research should focus on exploring these demographic influences through specific subgroup analyses and developing targeted interventions that consider these variabilities.

### 4.1. Limitations of the Study

This study has several limitations. Translating and adapting the K-10 and K-6 scales across different cultures may introduce inconsistencies, highlighting the need for rigorous local validation. Significant variability among the included studies, due to differences in design and sample sizes, affects the generalizability of the findings. The focus on English-language publications might exclude relevant studies in other languages, limiting comprehensiveness. Additionally, variability in sample sizes, from small groups to large cohorts, impacts the precision of reliability estimates. Despite being minimal, potential publication bias cannot be entirely excluded.

Moreover, it is important to acknowledge that this analysis did not include measures for validity testing. While reliability is a critical component of scale assessment, validity is equally important in determining whether the scales accurately measure psychological distress as intended. Future research should incorporate validity testing, such as construct, criterion, and content validity, to provide a more comprehensive evaluation of the K-10 and K-6 scales. This would ensure that these tools are not only reliable but also valid across different populations and cultural contexts.

### 4.2. Implications for Future Research

Future research should focus on validating these scales across diverse cultural contexts, including non-English publications, to reduce language bias. Standardizing methodological approaches can enhance consistency and precision in future studies. Examining how demographic factors influence reliability can help develop more tailored and accurate assessment tools. Despite an extensive search, some regional studies might not be captured due to database limitations. Future studies should ensure broader coverage by including more regional databases.

## 5. Conclusion

This meta-analysis confirms the high reliability of the Kessler Psychological Distress Scales (K-10 and K-6) across diverse populations and cultural contexts. The K-10 demonstrated excellent internal consistency, especially among adolescents and carers, while the K-6 also showed robust reliability across various settings. These findings highlight the effectiveness of the scales in measuring psychological distress globally, supporting their continued use in clinical practice and research. Despite some variability due to cultural and linguistic differences, the overall results emphasis the importance of these scales in providing reliable assessments of psychological distress, facilitating early intervention, and improving mental health outcomes worldwide. Future research should focus on further validating these tools across different settings to enhance their global applicability.

## Figures and Tables

**Table 1:** Summary of K-10 reliability generalization meta-analysis.

Total/subgroups	*k*	Estimate (*α*_+_)	*Z*val	90% CL	*Q*	*I* ^2^ (%)	*τ* ^2^
K-10 total	**26**	—	—	—	—	—	—
Random model	—	0.90	106.55⁣^*∗∗*^	[0.88, 0.91]	**955.23**⁣^*∗∗*^	**97.4**	**0.0014**
Population characteristics							
Random model	10	0.90	129.48⁣^*∗∗*^	[0.89; 0.92]	**110.13**⁣^*∗∗*^	**91.8**	**0.0003**
Adolescents	2	0.93	—	[0.92; 0.94]	—	—	—
Caregivers	1	0.91	—	[0.88, 0.94]	—	—	—
Educational	1	0.90	—	[0.85; 0.95]	—	—	—
General population	8	0.89	—	[0.89, 0.89]	—	—	—
Healthcare system	2	0.89	—	[0.85, 0.93]	—	—	—
Military	1	0.88	—	[0.87; 0.89]	—	—	—
Outpatients	2	0.88	—	[0.82; 0.94]	—	—	—
Drug users	2	0.89	—	[0.84, 0.94]	—	—	—
General adult population	4	0.91	—	[0.90,0 0.92]	—	—	—
General and psychiatric outpatients	2	0.93	—	[0.90, 0.92]	—	—	—
Language and adaptations							
Random model	12	0.90	141.90⁣^*∗∗*^	[0.89, 0.91]	**34.17**	**67.8**	**0.0002**
Arabic	1	0.88	—	[0.84, 0.92]	—	—	—
Danish	1	0.95	—	[0.91, 0.99]	—	—	—
English	1	0.89	—	[0.89, 0.89]	—	—	—
French	11	0.84	—	[0.75, 0.93]	—	—	—
Hindi	1	0.91	—	[0.88; 0.94]	—	—	—
Indonesian	2	0.89	—	[0.85; 0.93]	—	—	—
Japanese	1	0.91	—	[0.87, 0.95]	—	—	—
Korean	1	0.92	—	[0.89, 0.95]	—	—	—
Multiple South African Languages	1	0.89	—	[0.88, 0.90]	—	—	—
Persian	2	0.84	—	[0.89, 0.95]	—	—	—
Portuguese	1	0.89	—	[0.87, 0.91]	—	—	—
Continent							
Random model	7	0.90	93.78⁣^*∗∗*^	[0.88; 0.89]	**93.78**⁣^*∗∗*^	**99.0**	**0.0006**
Africa	—	0.85	—	[0.84, 0.86]	—	—	—
Asia	—	0.89	—	[0.87, 0.91]	—	—	—
Australia	—	0.90	—	[0.89, 0.91]	—	—	—
Europe	—	0.91	—	[0.90, 0.92]	—	—	—
North America	—	0.89	—	[0.88, 0.90]	—	—	—
North America and Australia	—	0.93	—	[0.93, 0.93]	—	—	—
South America	—	0.90	—	[0.89, 0.92]	—	—	—

*Note:* The table provides meta-analysis results for the Kessler Psychological Distress Scale (K-6) across various subgroups. The columns represent the number of studies (*k*), estimated reliability (*α*+), *Z*-value (*Z*val), 90% confidence limits (90% CL), *Q*-statistic (*Q*), heterogeneity (*I*^2^), and between-study variance (*τ*^2^). The random effects model indicates significant reliability estimates across subgroups with varying degrees of heterogeneity. Bold values indicate the total number of studies.

⁣^*∗∗*^Indicates statistical significance at *p* < 0.01.

**Table 2:** Summary of meta-analysis of K-6 reliability generalization.

Total/subgroups	*k*	Estimate (*α*_+_)	*Z*val	90% CL	*Q*	*I* ^2^ (%)	*τ* ^2^
K-6 total	**22**	—	—	—	—	—	—
Random model	11	0.84	392.55⁣^*∗∗*^	[0.87; 0.88]	**4219.24**⁣^*∗∗*^	**99.5%**	**0.00 82**
Population characteristics							
Random model	6	0.86	365.65⁣^*∗∗*^	[0.89; 0.89]	**580.90**⁣^*∗∗*^	**98.6%**	**0.0010**
Adolescents	2	0.82	—	[0.80, 0.84]	—	—	—
Drug users	3	0.84	—	[0.75, 0.93]	—	—	—
Educational	1	0.79	—	[0.78, 0.80]	—	—	—
General adult population	6	0.80	—	[0.80, 0.80]	—	—	—
General population	6	0.87	—	[0.86, 0.88]	—	—	—
General and psychiatric outpatients	1	0.85	—	[0.80, 0.90]	—	—	—
Healthcare system	3	0.85	—	[0.83, 0.87]	—	—	—
Immigrant	1	0.86	—	[0.84; 0.88]	—	—	—
Outpatients	1	0.89	—	[0.83, 0.95]	—	—	—
Language and adaptations							
Random model	9	0.84	331.55⁣^*∗∗*^	[0.79, 0.88]	**633.7**⁣^*∗∗*^	**98.7%**	**0.0038**
Arabic	1	0.88	—	[0.84, 0.92]	—	—	—
China	1	0.84	—	[0.83, 0.85]	—	—	—
English	11	0.85	—	[0.85, 0.85]	—	—	—
Farsi	1	0.80	—	[0.72, 0.88]	—	—	—
French	1	0.84	—	[0.75, 0.93]	—	—	—
Indonesian	1	0.83	—	[0.78; 0.88]	—	—	—
Japanese	2	0.90	—	[0.89, 0.91]	—	—	—
Korean	1	0.89	—	[0.86, 0.92]	—	—	—
Multiple South African Languages	3	0.70	—	[0.69, 0.71]	—	—	—
Continent							
Random model	6	0.86	371.51⁣^*∗∗*^	[0.86; 0.87]	**23.02**⁣^*∗∗*^	**87.0%**	**0.0004**
Africa	2	0.81	—	[0.78; 0.84]	—	—	—
Asia	4	0.87	—	[0.86; 0.88]	—	—	—
Australia	3	0.85	—	[0.84; 0.86]	—	—	—
North America	2	0.86	—	[0.85; 0.87]	—	—	—

*Note:* The table provides meta-analysis results for the Kessler Psychological Distress Scale (K-6) across various subgroups. The columns represent the number of studies (*k*), estimated reliability (*α*+), Z-value (*Z*val), 90% confidence limits (90% CL), *Q*-statistic (*Q*), heterogeneity (*I*^2^), and between-study variance (*τ*^2^). The random effects model indicates significant reliability estimates across subgroups with varying degrees of heterogeneity. Bold values indicate the total number of studies.

⁣^*∗∗*^Indicates statistical significance at *p* < 0.01.

**Table 3:** Summary of combined K-10 and K-6 meta-analysis.

Scales	Mean Cronbach's *α*	SD	*t*-Value	*p*-Value	95% CI (mean difference)	Cohen's *d*	95% CI (Cohen's *d*)
K-10	0.89	0.04	2.5036	0.01836	[0.0095, 0.0945]	0.77	[0.17, 1.35]
K-6	0.84	0.09					

*Note:* The table presents the comparative meta-analysis results for the Kessler Psychological Distress Scales (K-10 and K-6). The mean Cronbach's *α*, standard deviation, *t*-value, *p*-value, 95% confidence interval for the mean difference, Cohen's *d*, and 95% confidence interval for Cohen's *d* are provided. The analysis shows that the K-10 scale has a higher reliability coefficient compared to the K-6 scale, with the difference being statistically significant (*p*=0.01836) and practically significant (Cohen's *d* = 0.77).

## Data Availability

The data that support the findings of this study are available from the corresponding author upon reasonable request.
